# T-Cell Immune Responses Against Env from CRF12_BF and Subtype B HIV-1 Show High Clade-Specificity that Can Be Overridden by Multiclade Immunizations

**DOI:** 10.1371/journal.pone.0017185

**Published:** 2011-02-18

**Authors:** Daniela C. Mónaco, Ana M. Rodríguez, María F. Pascutti, Mauricio Carobene, Juliana Falivene, Alejandro Gómez, Cynthia Maeto, Gabriela Turk, José L. Nájera, Mariano Esteban, M. Magdalena Gherardi

**Affiliations:** 1 Centro Nacional de Referencia para el SIDA, Universidad de Buenos Aires, Buenos Aires, Argentina; 2 Departamento de Biología Molecular y Celular, Centro Nacional de Biotecnología, CSIC, Campus Universidad Autónoma, Madrid, Spain; The University of Chicago, United States of America

## Abstract

**Background:**

The extreme genetic diversity of the human immunodeficiency virus type 1 (HIV-1) poses a daunting challenge to the generation of an effective AIDS vaccine. In Argentina, the epidemic is characterized by the high prevalence of infections caused by subtype B and BF variants. The aim of this study was to characterize in mice the immunogenic and antigenic properties of the Env protein from CRF12_BF in comparison with clade B, employing prime-boost schemes with the combination of recombinant DNA and vaccinia virus (VV) vectors.

**Methodology/Principal Findings:**

As determined by ELISPOT from splenocytes of animals immunized with either EnvBF or EnvB antigens, the majority of the cellular responses to Env were found to be clade-specific. A detailed peptide mapping of the responses reveal that when there is cross-reactivity, there are no amino acid changes in the peptide sequence or were minimal and located at the peptide ends. In those cases, analysis of T cell polifunctionality and affinity indicated no differences with respect to the cellular responses found against the original homologous sequence.

Significantly, application of a mixed immunization combining both clades (B and BF) induced a broader cellular response, in which the majority of the peptides targeted after the single clade vaccinations generated a positive response. In this group we could also find significant cellular and humoral responses against the whole gp120 protein from subtype B.

**Conclusions/Significance:**

This work has characterized for the first time the immunogenic peptides of certain EnvBF regions, involved in T cell responses. It provides evidence that to improve immune responses to HIV there is a need to combine Env antigens from different clades, highlighting the convenience of the inclusion of BF antigens in future vaccines for geographic regions where these HIV variants circulate.

## Introduction

More than twenty-five years have passed since the human immunodeficiency virus (HIV), the causative agent of acquired immunodeficiency syndrome (AIDS), was isolated and identified. But, although the development of antiretroviral drugs has been very successful, an efficient vaccine is still needed to confront and finally knock down the devastating epidemic. One of the challenges to be addressed and ultimately overcome when developing a vaccine is the high variability of HIV-1, implying both intra- and inter-subtype variation. This genetic capacity allows the virus to escape from the host immune system and also hinders predictions for vaccine composition.

The M group of HIV-1, responsible for the pandemic, has been differentiated in nine subtypes (A–K) and two sub-subtypes, A2 and F2 [1], [2]. Moreover, the complexity of the epidemic has been largely elevated with the dissemination of circulating recombinant forms (CRFs) with a defined genetic structure. Currently, up to 48 CRFs have been described (http://www.hiv.lanl.gov/content/sequence/HIV/CRFs), and considered responsible for 18% of the infections [2], [3]. Inter-clade differences can be up to 35% in the *env* region, and although there are several studies which clearly indicate cross-clade-reactive HIV-1-specific CD8+ T-cell responses [4], [5], [6], [7], several data demonstrates that highly specific T-cell receptors can be sensitive to single amino acid (aa) changes [8], [9]. In this sense, escape from existing T-cell responses in infected individuals by single mutations in epitopes [10], [11] largely demonstrate this concept.

A major obstacle to the development of an HIV vaccine is the lack of knowledge about the precise correlates of protection. Nevertheless, it is accepted that balanced humoral and cellular immune responses are required [12]. A highly promising strategy for the induction of strong antigen-specific responses is the combination of different vectors (especially DNA and viral vectors) for delivering genetic immunogens in prime/boost approaches.

In this regard, the results of the last preventive phase III Thai trial with a combination of a poxvirus vector (canarypox) and a recombinant protein gp120 for different clades (CRF01_AE, B), while revealing modest efficacy represent an injection of optimism for the vaccine development study area [13]. One of the topics of relevance to be analyzed is the ability of heterologous prime-boost immunization protocols to induce specific T-cell immune responses capable of recognizing multiple HIV-1 variants.

The AIDS epidemic in South America is caused by multiple HIV-1 subtypes including subtypes B, F, and C, in addition to BF and BC recombinant forms. In Argentina, epidemiological studies revealed that the early predominance of subtype B has been diminished by the emergence of BF recombinants [14], [15], [16], [17], and that the BF epidemic comprises the widespread of CRF12_BF and several unique recombinant forms (URFs) with a CRF12-related structure [18]. Recent phylogenetic studies showed for the first time that CRF12_BF viruses spreading in Argentina and Uruguay constitute a single epidemic with evidences of multiple genetic exchanges among countries [19]. Even more, although in a minor proportion, some cases of BF recombinant viruses related to CRF12_BF have also been reported in other countries as Bolivia [14], Venezuela [20], [21], Chile, Spain [21],[22] and Paraguay [23]. All these epidemiological data highlight the importance of CRF_12BF and BF variants especially in South America.

The extreme genetic diversity of the HIV-1 envelope (Env) poses a daunting challenge for the generation of an effective HIV/AIDS vaccine, being Env the principal target for HIV-1-specific antibody responses, which also serves as a potent T-cell immunogen. With regard to the epidemic in Argentina, it must be pointed out that differences between EnvB and EnvBF (from CRF_12BF) sequences varied from 23.7 to 26.5%.

We have recently reported the characterization of DNA and MVA vectors that express Nef from HIV-1 CRF12_BF, describing their capacity to induce a high immune specificity with low cross-reactivity against Nef from subtype B [24]. In this study we have extended that analysis expressing a synthetic form of Env CRF_12BF from DNA and Vaccinia virus vectors as a model to evaluate the EnvBF immunogenicity characteristics after prime-boost immunizations.

## Results

### 1. Construction and characterization of DNA and VV vectors expressing EnvBF

A recent study from our research team reported the highly immunogenic specificity induced by Nef from CRF12_BF (NefBF) when it is delivered from DNA and MVA vectors [24]. Continuing with this research line we have constructed DNA and VV vectors (on a Western Reserve WR backbone) that express a syngp160 protein from CRF12_BF, to note is the fact that the genetic composition of Env from this CRF is merely of subtype F [14]. An evaluation of the correct expression of the EnvBF protein was done by Western blot and confocal microscopy as part of the characterization of the vectors constructed using different cell lines.

**Figure 1:**
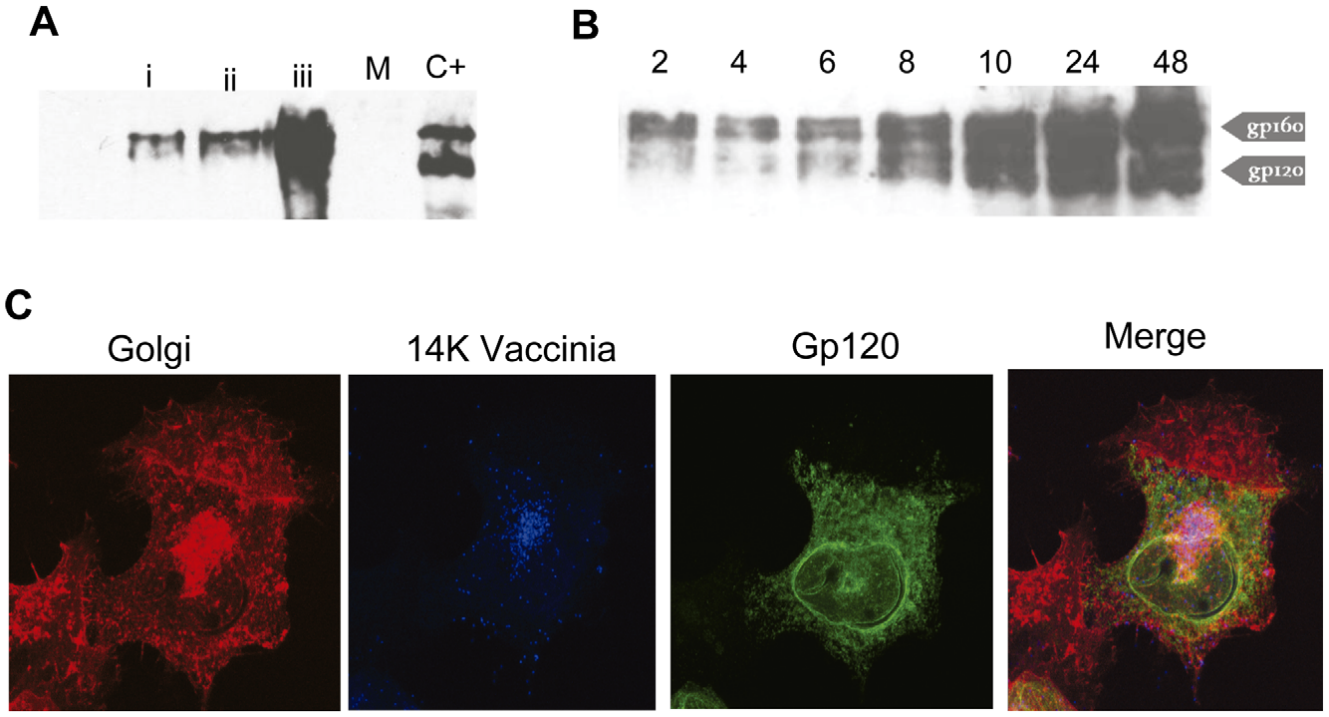
Characterization of the DNA and VVEnvBF vectors generated. **A**) The expression of Env was visualized by Western Blot in cell extracts obtained 48 hrs post-transfection of 293-T cells with p*env*BF or p*empty* plasmids. Lanes i to iii were loaded with 5, 10 and 20 µg of total protein. Mock transfected cells (M) and cell extracts infected with the VVEnvB virus (C+) were employed as negative and positive controls. **B**) **i-** Time-course expression of EnvBF after infection of BSC-40 cells. At the indicated hours post-infection Env was detected by WB in pellet samples of infected cells at 5 PFU/cell. **C**) Immunofluorescence analysis of EnvBF expression after VVEnvBF infection (0.1 PFU/cell) of HeLa cells. After 18 hrs p.i., cells were fixed, permeabilized, and incubated with polyclonal anti-Env antibody to show Env (green), with C3 monoclonal antibody against 14 Kda VV protein (blue) or with antibody against the wheat germ antigen, to show Golgi (red). To the right is the colour merging.

The expression of the DNA vector evaluated after 48 h by transfection of 3T3 cells depicts by Western blot a protein with a molecular weight of 160 KDa (Figure 1A); the intensity of the band augmented with the quantity of protein loaded. Env expression from the rVV*env*BF was evaluated at different times p.i in BSC40 cells. As shown in Fig. 1B, gp160 is observed as early as 2 hrs pi, increasing with time of infection. Both the complete 160 kDa and processed 120 kDa products were observed. Similar results were found when the same kinetics was repeated in the murine 3T3 cell line (data not shown). After infecting HeLa cells, intracellular localization of Env BF, as analyzed by immunofluorescence with Env specific antibodies, was predominantly found in the cytoplasm and concentrated in the Golgi apparatus as it can be expected from a protein that is glycosylated and secreted (Fig. 1C). In blue, the specific staining for the envelope VV 14 k protein (A27L) is shown.

### 2. Immunogenicity of EnvBF vs EnvB proteins: cellular responses induced after DNAprime/VVboost vaccine regimes

#### 2.1 Reactivity against subtype B peptides

**Figure 2:**
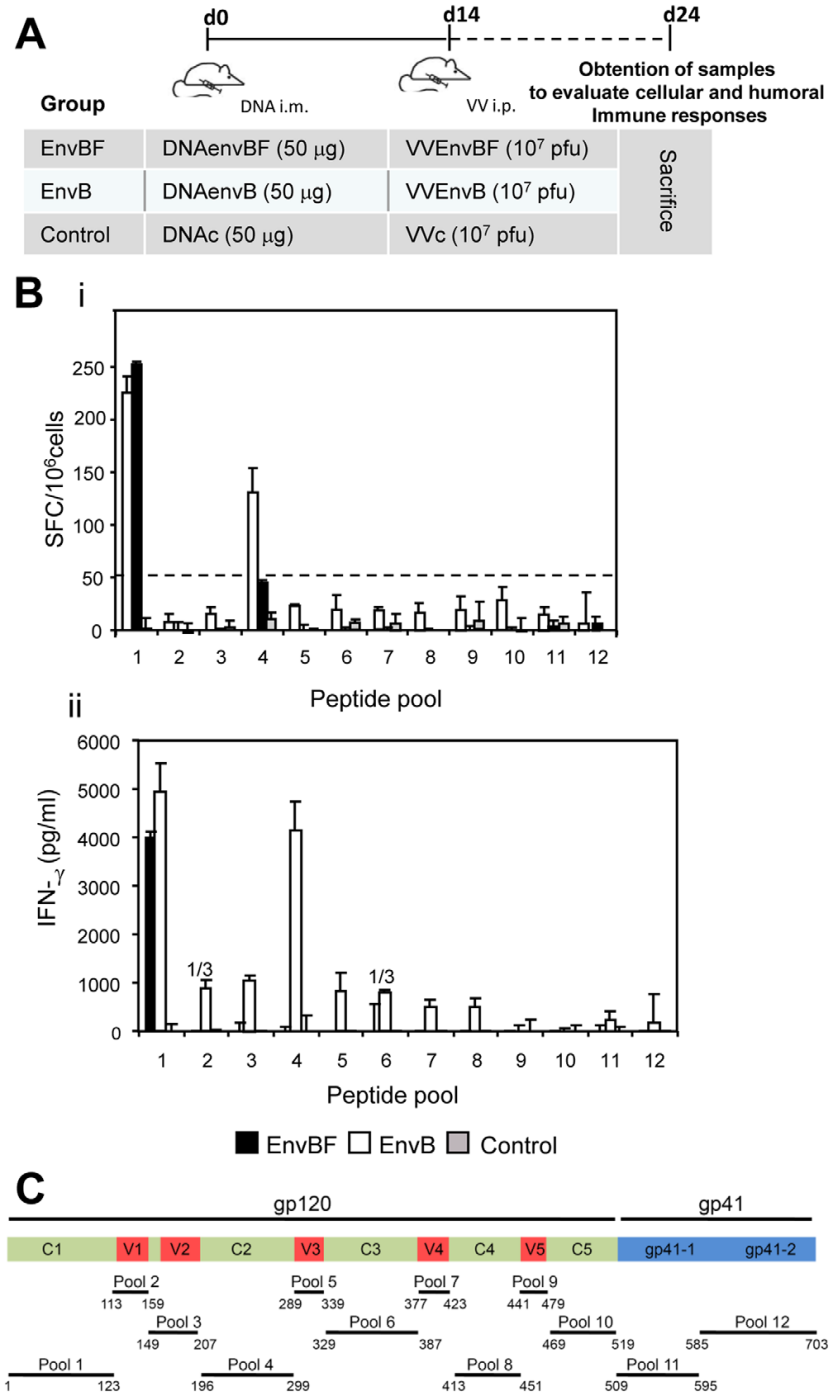
Immunogenicity of EnvBF vs EnvB proteins: cellular responses induced after DNAprime/VVboost vaccine regimes. **A**) Description of the immunization scheme applied in groups of four Balb/c mice. **B**) Ten days after the last immunization dose, cellular immune responses against EnvB were evaluated by ELISPOT (i) or ELISA (ii). To this, splenocytes from mice of the different groups were restimulated with the Env Con B peptide pools of indicated, during 24 hrs (i) or 72 hrs (ii). Bars represent the average net number of spots +/− SD for triplicate wells of pooled splenocytes (i), or IFN-γ specific levels found in supernatants after substracting 2× value found in control unstimulated cultures (ii). **C**) Scheme indicating the gp160 region included in the different peptide pools used, which were numbered from 1 to 12. Numbers upper the bars: 1/3 indicated that in one out of three experiments a positive value was obtained. Data are representative of three independent experiments.

After showing correct expression of gp160 from the DNA and VV vectors expressing EnvBF, next we evaluated their immunogenicity in a Balb/c mouse model. For this, four mice per group were immunized according to the protocol depicted in Figure 2 A.

Specific responses were evaluated by stimulating the splenocytes of each group with pools of EnvB peptides representing the constant and variable regions of gp120 and a portion of gp41 (see detailed scheme in Fig. 2 C). Determinations were performed by ELISPOT assay (Fig. 2Bi), or quantifying the levels of specific IFN-γ secreted in the cell cultures (Fig. 2Bii). The ELISPOT assay of mice that received the EnvB immunization revealed that the main cellular reactivity was directed against pool 1 (corresponding to the C1 region) (Fig. 2C) and pool 4 (C2 region). Whereas in those mice which received the EnvBF immunization schedule, only reactivity against pool 1 (C1 region) was detected, indicating cross-reactivity at this region. Magnitude of the responses against this region (C1) did not differ significantly between both groups (p>0.05) (Fig. 2i). By quantifying by Elisa the levels of specific IFN-γ secretion after a longer incubation period (72 hrs) of stimulation with the same pools of peptides, we were able to detect positive reactivity against many regions of the protein (Fig. 2Bii), but this response was subtype specific as it was only detected after the EnvB immunization.

#### 2.2 Mapping the gp160B-specific response

**Figure 3:**
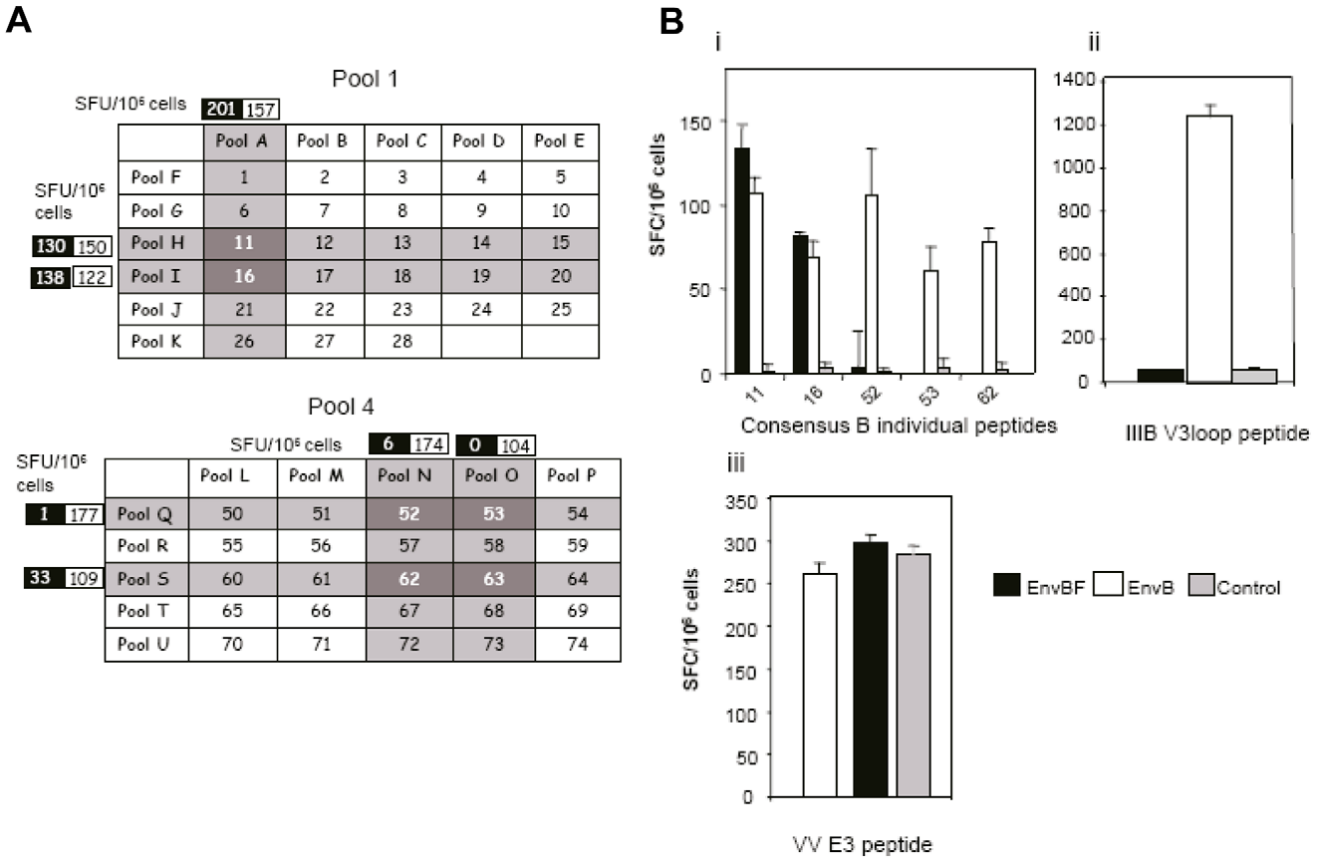
Mapping the gp160B specific response. **A**) Matrix-peptide based analysis for poo1 and pool 4 corresponding to the constant C1 and C2 regions of gp160 EnvB protein. Number of SFU/10^6^ cells found in EnvBF and EnvB immunized mice are indicated in black and white boxes respectively, and results found in reactive pools are shown. **B**) Reactive peptides identified in A were evaluated in experiments in which single individual EnvB peptides were employed in the ELISPOT assays. Figure shows magnitude of the responses detected against consensus B identified peptides (i), the IIIB CD8^+^ V3 loop peptide (ii), and the VV CD8^+^ E3 peptide (iii). Results are representative of three independent experiments.

To further determine which peptides from pools 1 and 4 of the EnvB protein were the targets of the response detected in Figure 2Bi, a matrix for these two regions was designed. Figure 3A describes the matrix for pools 1 and 4 (C1 and C2 regions of EnvB respectively) and the number of SFC/10^6^ cells detected in experiments in which the same scheme depicted in Fig. 2A was applied. This figure highlights the positive responses found in EnvB and EnvBF groups, responses for the control group were below 30 SFC/10^6^ for all the matrix pools. As it can be seen for the matrix of pool 1, peptides 11 and 16 were reactive. And in the case of pool 4, positive responses indicated that peptides 52 and 53 (consecutive peptides, therefore representing a unique epitope) and 62 and 63 (also consecutive) were the target of the responses found in the EnvB immunized group. Peptides identified in these matrix arrangements were confirmed by ELISPOT assays in which the individual peptides were employed to stimulate the cells (Fig. 3B). In these assays two additional predefined peptides were included: such as the CD8^+^ peptide p18IIIB-I10 (RGPGRAFVTI) from the IIIB V3 loop, (immunodominant and restricted for H-2D^d^ presentation), and the CD8^+^ peptide from VV: E3, that derives from the viral gene E3L, (inhibitor of the antiviral state induced by interferons) [25]. After EnvB immunization the response found against the p18IIIB-I10 peptide (Fig. 3B ii) was subtype specific and showed a magnitude in concordance with that previously described for this model of immunization [26]. It must be highlighted that in this group, the corresponding peptide of the EnvB consensus set was not recognized.

As it can be expected from the VV E3 peptide (Fig. 3B iii) a similar level of response was detected in the three groups, suggesting that a comparable level of immunogenicity was triggered in the different groups of mice.

#### 2.3 Reactivity against subtype BF peptides

**Figure 4:**
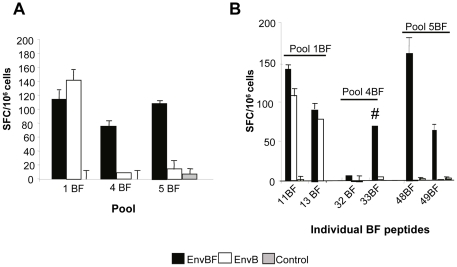
Reactivity against subtype BF peptides. **A**) Cellular immune responses against the EnvBF regions indicated (pools 1, 4 and 5). **B**) After performing a matrix-peptide based analysis as it is depicted in Fig. 3A, the identified BF peptides were evaluated individually. Data are representative of two independent experiments. Symbol: # denotes that the response shown was obtained in 50% of the experiments.

Our next aim was to evaluate the immune response induced against peptides of certain regions of the gp160BF protein. Different groups of animals received the same DNA/VV immunization scheme previously described, and after 10 days of the last immunization we evaluated the specific cellular immune response. For this, synthetic peptides of 15 aa with an overlap of 11 aa comprising the C1 (pool 1), C2 (pool 4), and V3 (pool 5) regions based on the CRF12_BF sequence (identical to that expressed from the BF vectors) were employed (see Materials and Methods). A shown in Fig. 4A, both EnvB and EnvBF groups recognized pool 1 BF (C1 region), and the magnitude of the responses found did not differ significantly between both groups. This scenario is similarly to what happened when stimulating cells with the corresponding pool1B (Fig. 2Bi). On the other hand, significant specific cellular responses directed to pools 4BF and 5BF (C2 and V3 regions) were only detected after immunization with vectors expressing the homologous antigen (gp160BF protein). The cellular immune response against gp160BF was mapped by a matrix peptide based analysis as described in Figure 3. Thus, after the identification of the peptides targeted doing a matrix ELISPOT repeated in two different experiments (data not shown), the specific responses employing the individual peptides as the antigenic stimulus were confirmed (Fig. 4B). Peptides 11BF and 13BF accounted for the response detected against the C1 region. While in the C2 region (pool 4BF) two consecutive peptides, 32 and 33, were identified by the matrix ELISPOT, confirming the positive response against the individual peptide 33BF. This peptide seems to be subdominant as we could only detect a positive response (slightly above the detection limit) in two out of four experiments (two evaluations with matrix peptide pools and two with individual peptides). Moreover, we obtained a response specific for 33BF peptide of a magnitude of 90 SFC/million when animals received a boost dose of the VVEnvBF four times higher (4×10^7^ PFU/animal), in which case the responses evaluated against the other peptides were saturated (non-countable) (data not shown). Finally, the matrix of the V3 region (pool 5BF) led to the identification of the 48BF peptide, confirming these responses with the individual peptide (Fig. 4B).

#### 2.4 Fine mapping of responses: analysis of the sequences and localization of the B and BF peptides targeted

**Figure 5:**
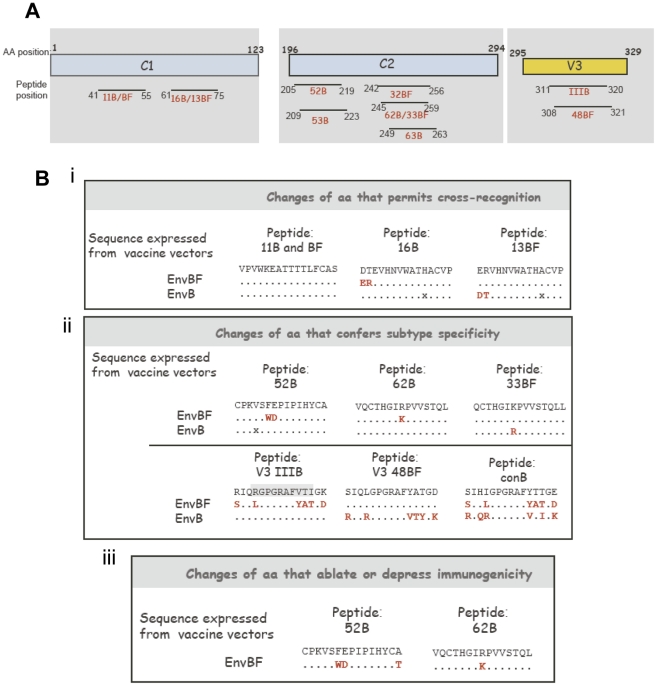
Analysis of the sequences and localization of the B and BF peptides targeted. **A**) Scheme showing the different Env regions and localization of the B and BF peptides targeted after the EnvB and EnvBF immunizations **B**) Description of the aa sequence of the peptides recognized and of the sequence expressed from the vectors Description of the aa changes that permits cross-recognition i), that confer suptype specificity ii) and of the changes that ablates or depress immunogenicity iii) An x indicates that for that position a different aa is encoded from the DNAenvB plasmid.

After the identification of the peptides that were induced by Env immunizations based on the B and BF subtypes, we proceeded to map the immunogenic peptides targeted. Fig. 5A shows their localization within the structural regions of the protein. Thus, in the C1 region two zones were targeted, one from aa 41 to 55 (peptide 11B and BF) and the other from aa 61 to 75 (peptides 16B and 13BF).

In the C2 region the two immunogenic regions identified span from aa 205 to 223 (peptides recognized: 52B, 53B) and from aa 245 to 263 (peptides recognized: 62B, 63B, 32BF and 33BF). As expected, a unique region was identified inside the V3loop, covering aa 308 to 321 (peptides recognized: IIIB and 48BF).

Fig. 5B describes the sequence analysis of the peptides targeted, highlighting the aa changes that permit cross-reactivity (i), that confer subtype specificity (ii) and those that ablate or diminish immunogenicity (iii). For the first instance (i), it can be seen that in the case of peptide 11 no aa changes occurred between both sequences (envB and envBF), or as it occurred for peptide 13BF and 16B, aa changes were placed at the left end of the peptides. On the contrary, when we analyzed the cases for which we found subtype specificity (Fig. 5B ii), the aa changes were located on the centre of the peptide (peptides 52B, 62B and 33BF on C2), affecting the recognition site by the TCR. The analysis of the aa changes that occurred on the V3 region (Fig. 5Bii, lower panel) indicated that a great number of changes were observed in this region (in agreement to be a highly variable region) explaining the high subtype specificity observed (we could only detect the specific responses with the clade-matched peptide, (i.e.: IIIB with IIIB, but not with the consensus B peptide). The analysis of aa changes that ablate or diminish immunogenicity (Fig. 5B iii), revealed that within the C2 region the substitution of Phe- Glu (FE) in the B sequence for Trp-Asp in the BF (sequence of 52B with respect to its counterpart in BF), ablated the immunogenicity. In the case of the region where peptides 62B and 33BF are located, the change of Lys (K) in the EnvB sequence by Arginine (R) in the EnvBF sequence notably reduced its immunogenicity (see description of Fig. 4B).

### 3. Functional avidity of T-cell responses showing cross-clade recognition

Results of the experiments described above lead us to the conclusion that after the immunizations based on vectors expressing Env from subtypes B or BF, the majority of the peptides recognized were suptype-specific, except for the two peptides targeted corresponding to the C1 region, for which we found cross-recognition. As observed in the previous section, one of them (peptide 11) has the same sequence for both B and BF *env* sequences. But unlike the other peptides (16B or 13BF), sequences were not identical since they differ in two aa located at the left end.

Functionality of T-cell responses in terms of quality or capacity to secrete multiple cytokines has been shown critical in relation to the protection capacity of the T-cell response [27]. Therefore, we considered of interest to compare the cytokine pattern of the T-cell responses subtype-specific versus cross-clade reactive. For this, splenocytes from mice obtained ten days after the booster with EnvB or EnvBF vectors as depicted in Fig. 2, were assayed by ICS following a 72 hrs of stimulation with the pools 1 and 4 (C1 and C2) of the EnvB sequence. Thus, for the EnvBF immunized group, responses against C1 implied cross-recognition. First of all, the ICS analysis permits us to determine whether the response was mediated by CD4+ or CD8+ T cells, finding that for both pools CD4+T cells were mediating the specific response found. In the case of the pool 1, we confirmed the CD4 phenotype after the stimulation with the individual peptides.

**Figure 6:**
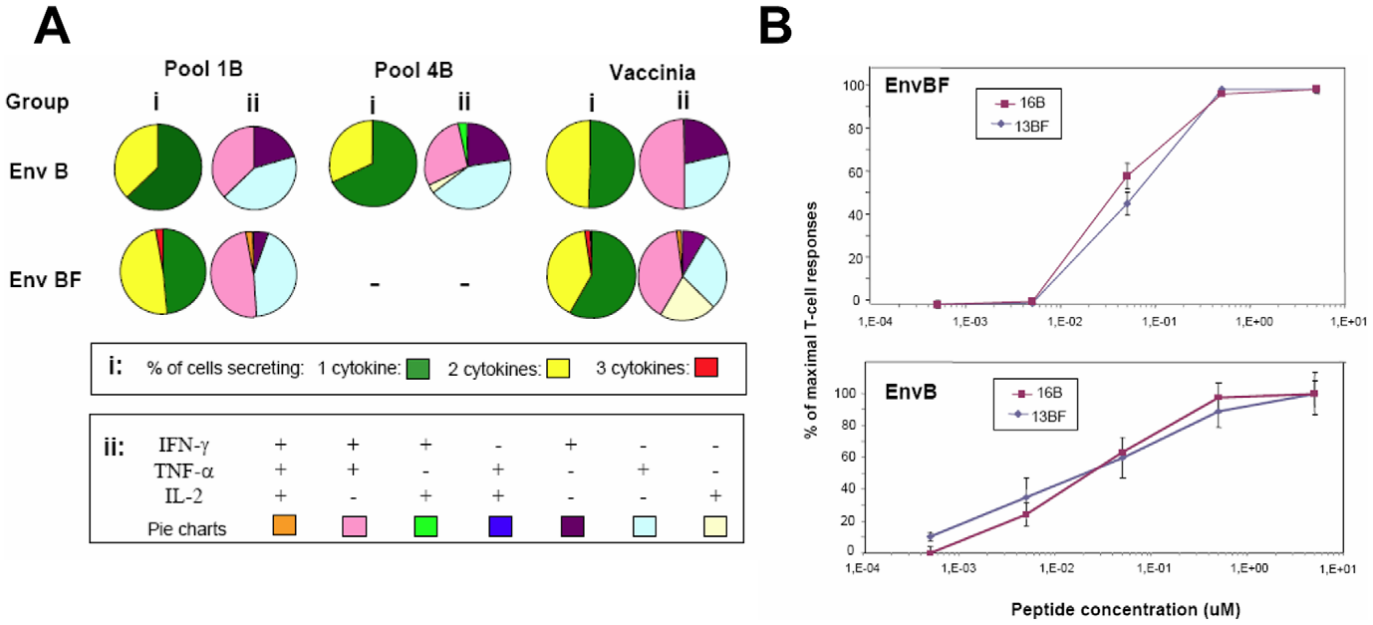
Functional and avidity characteristics of T-cell responses with cross-clade recognition. **A**) Quality characterization of T cell responses against Pool 1 and Pool 4 consensus EnvB peptides and against VV antigens, in EnvB and EnvBF immunized mice. Ten days post boost immunization splenocytes were harvested and the percentage of antigen specific CD3+ CD4+ T cells producing IFN-γ, TNF-α, or IL-2 was quantified by flow cytometry. i) Data shows the percentage of CD3+ CD4+ T cells secreting one, two or the three cytokines ii) Distribution of the cytokine response comprising the different cell populations producing IFN-γ, TNF-α, and IL-2 individually or in combination within CD3+ CD4+ T cells. **B**) T cell functional avidity is defined as the concentration required to achieve half-maximal recognition of the wild-type peptide (EC_50_). Ten days after the boost immunization splenocytes from immunized mice were assayed by Elispot against serial dilutions of 13BF and 16B peptides. Data represents the percentage of the maximal response (net number of SFU/million of cells stimulated with a peptide concentration of 5 µM). Results shown are representative of two independent experiments.

In Fig. 6A it can be seen that the quality of the CD4+ T cell response against the peptides of pool 1 were similar for both groups. Moreover, in EnvBF immunized mice in which cross-reactivity is displayed, we could find at least a minimal proportion of cells (2,8%) producing the three cytokines simultaneously. Thus, the proportion of mono and bi-functional cells detected in B and BF groups accounted for 62,5 and 48,6% (one cytokine) and 37,5% and 48,7% (two cytokines) of the responses respectively. Responses against peptides of pool 4 could be only evaluated in the B group, where a similar pattern to that detected against pool 1 was found. When the quality of the specific CD4+ T cell responses against VV antigens was analyzed, a similar pattern was found for both groups except that IL-2+ responses were only detected for the BF group.

T cell functional avidity defined as the capacity of the specific cells to recognize its specific antigen at lower concentrations is a reflection of the efficiency of the effector cells. During HIV infection, functional avidity of both CD8+ and CD4+ T cells were found to be incremented in HIV controller subjects [28], [29], [30]. To investigate if the cross-clade recognition due to the aa changes found in 16B/13BF may affect the avidity pattern of the responses, we analyzed functional avidity against these peptides (differing in two aa at the left ends). ELISPOT assays at different peptide concentrations were performed, with the aim to define if the recognition pattern differs between homologous or heterologous responses. In Fig. 6B it can be seen that the curves obtained for both peptides were similar, showing no differences between the functional activities. Thus, values for 50% of the maximal T-cell responses (EC_50_) did not differ significantly between homologous vs heterologous responses (16B vs 13BF), independently of the immunized group, thus EC_50_ values calculated with a sigmoid dose-response curve (GraphPad, software) were of 0,014 (13BF) and 0,01 uM (16B) for both immunized mice groups (EnvBF or EnvB).

### 4. A mixed immunization regime combining both clades (B and BF) induced a broad T- cell response, covering the main peptides targeted after the single clade immunizations

**Figure 7:**
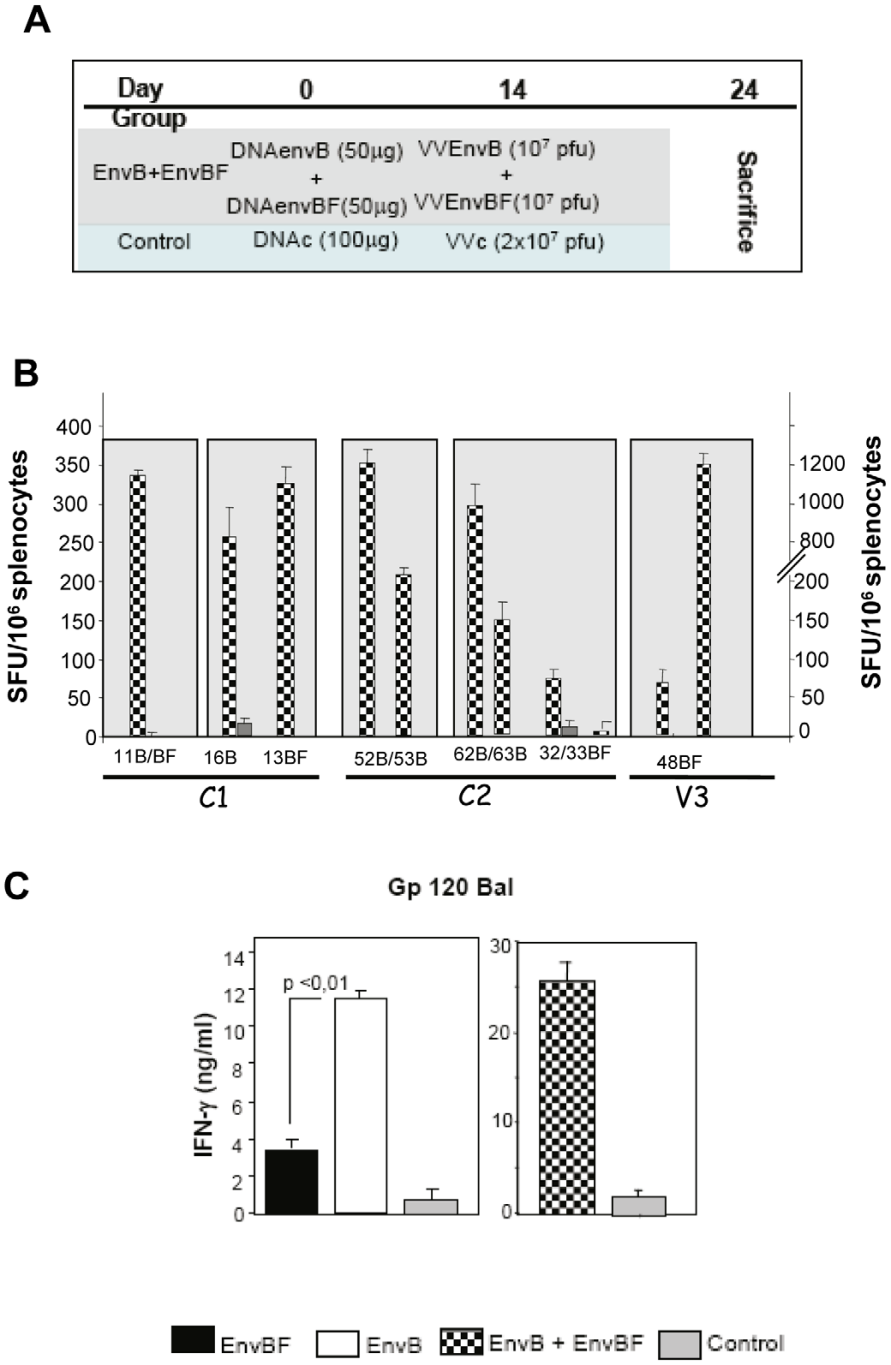
A mixed immunization regime combining both clades (B and BF) induced a broad immune response. **A**) Immunization scheme describing viral and DNA doses applied. **B**) Ten days after the boost immunization, T cell response induced after the mixed immunization was analyzed against the different B and BF peptides targeted after the single-clade immunizations. Peptides were grouped according to its localization within each protein region; #: indicates that positive responses were found in one of two experiments. **C**) Ten days after the boost, splenocytes from mice immunized as its is depicted in A or in Figure 2A were stimulated with gp120 Bal (1 µg/ml) during 72 hrs and afterwards IFN-γ levels in the supernatant were quantified.

The results described in the previous sections clearly demonstrated that the immunization schemes applied based on EnvBF or EnvB immunogens induced highly clade-specific responses. Therefore, our next aim was to evaluate if it is possible to induce a broad response after the application of a mixed immunization schedule implying a multi-clade formulation combining the vectors that express the B or BF antigen. To this, two groups of 4 mice were immunized as it is depicted in Fig. 7A, where it can be seen that in the dual immunized group the doses of DNA and VV vectors of each clade were equivalent to those administered in the single-clade schemes (view Fig. 1). After ten days, we evaluated the cellular immune responses against the individual B and BF peptides previously identified to be targeted after the immunizations based on the single clades. We found that the mixed immunization regime induced a broad response covering the majority of the peptides targeted after the single-clade schemes. In Fig. 7B it is shown the cellular response detected against the B and BF peptides located in the specified regions of the protein. With respect to the C1 region, all the peptides previously identified were targeted, peptide 11 (present in B and BF), and both 16B and 13BF were recognized as it occurs after the single clade immunizations. On the other hand in the C2 region where clade specific responses were previously detected, here after the dual immunization, the sequences corresponding to 52/53B and 62/63B peptides were targeted, and with respect to the 32/33BF peptides the response found also show a subdominant pattern (low magnitude and positive responses in 50% of the experiments) as it occurs after the single BF immunization.

After the BF regime we have detected a strong response against the V3 loop BF peptide (48BF) comparable to that generated against peptide 11 (see Fig. 4B). But after the application of the mixed immunization in which it is also present the IIIB V3 region, immunodominant for the H2D^d^ haplotipe, the response against the 48BF peptide was significantly diminished, being slightly above the cut-off limits established (67,5±15 SFC/mill). Therefore, when we compared the response detected against 48BF in both types of experiments (BF single-clade vs, dual immunization) in relation to that obtained against the 11 peptide (48BF specific SFU/11 specific SFU) we found that this value was of 1 and 0.2 after the BF and dual immunization schemes, respectively.

### 5. Cellular and humoral immune responses against recombinant gp120 protein of subtype B

We further explored the capacity of the cellular immune responses induced after the three schemes applied (single versus mixed immunizations), evaluating levels of IFN-γ secreted in the presence of rgp120 BAL (subtype B) after the stimulation of the cells during three days in culture (Th cell activation principally). As it can be expected following the EnvB immunization, a strong response against a homologous gp120 protein was detected, whereas after the BF immunization a minor response was found (Fig. 7C). The level of the cross-reactivity detected after this immunization scheme varied from 3% to 54% of the maximal response, depending on the experiment, found after the homologous immunization. On the other hand, after the mixed vaccination the strong response against gp120 subtype B was maintained (Fig. 7C right panel).

Serum humoral immune responses induced after 10 days of the different immunization schemes applied (Fig. 1A and Fig. 7A) were analyzed by ELISA, evaluating Ab response against gp120 from subtype B (gp120 BAL and gp120 IIIB), as no recombinant gp120 from BF is available. In Fig. S1 it can be seen that mice from the EnvB group showed high levels of IgGs that recognized both types of gp120 from subtype B, whereas the EnvBF immunization generated higher levels of IgGs showing cross-reactivity against gp120 BAL with respect to those exhibited against gp120IIIB. Following the dual immunization, Ab levels against BAL protein were higher than those against IIIB, following a pattern similar to that found after the EnvBF immunization scheme.

### 6. Recognition of EnvBF and EnvB during natural HIV-1 infection

To have an approximation of what could be the scenario of the reactivity of the cellular immune response against Env during the natural HIV infection, we analyzed the Env-specific response in a subgroup of HIV-infected persons from a cohort of individuals enrolled during seroconversion to HIV and under longitudinal follow-up. The great advantages of an immune response analysis during this time of the infection compared to the chronic phase, is that a lesser grade of virus variation might have occurred. Also, the host immune system is best preserved with lower levels of immune activation and T-cell exhaustion. The main characteristics of the HIV infected PBMCs donors used in the Elispot assay are described in Fig. S2A. We performed the assay employing the peptide pools corresponding to the constant and variable Env B and BF regions that were target of the response after mice immunization (C1 and C2 and V3). As expected, the most recognized peptide pools were those of the constant regions (positive responses against V3B and V3BF were only found in two individuals with lower magnitudes). Out of sixteen samples analyzed, six exhibited a positive response against Env. For these cases, when the total response was evaluated (as the sum of the partial responses against each of the env regions) a cellular response with marked subtype specificity was found, as four out of six were clearly subtype specific with respect to the infecting virus (patients 391, 834, 126 and 732). And in cases with comparable levels of cross-reactivity between B and BF peptide pools (patients 690 and 183), a higher significant response was found towards the homologous subtype (patient 690). These results suggest that during the primary HIV infection, there is a tendency to recognize Env peptides homologous to the subtype of the infecting virus, in agreement with the results described above after mice immunization.

## Discussion

The hypervariability of HIV generating a high rate of antigenic heterogeneity among the different virus variants circulating throughout the world poses a critical obstacle to vaccine development. Several strategies are currently being explored to circumvent this challenge, one of them is to design vaccine strategies based on multivalent formulations including antigens of the main subtypes circulating in a certain region.

Molecular epidemiological studies showed that, with the exception of Sub-Saharan Africa, where almost all subtypes, CRFs and URFs have been detected, there is a specific geographic distribution pattern of HIV-1 subtypes [31], [32]. The HIV epidemic in Argentina is primarily dominated by infections caused by B and BF recombinant variants related to the CRF12_BF.

In previous studies from our laboratory we characterized the immunogenicity of Nef from CRF12_BF and BF variants, evaluating the characteristics of the cellular immune response in primary HIV infected persons [33], and in a murine model expressing the protein from DNA and MVA vectors [24]. In the latter study we found high subtype specificity, generating low levels of cross reactivity after an expanded immunization schedule. Now we have analyzed in a mouse model the immune characteristics with epitope mapping of EnvBF in comparison with EnvB, by expressing the Env protein from DNA and VV vectors. Mapping of the peptides targeted after a DNA/VV protocol, showed that after EnvB immunization a total of 5 peptides (overlapping peptides were not considered) were recognized, of which two were located inside the C1 region (aa41 to 56 and aa61 to 75), other two in the C2 region (aa205 to 223 and aa245 to 259) and the fifth one corresponded to the previously characterized CD8 V3 loop epitope (aa311 to 320). On the other hand, after the EnvBF immunization schedule performing the characterization of the gp160 regions that resulted immunogenic following the B schedule, we found that four BF peptides were targeted.

We identified the two peptides confined in the C1 region as CD4+, in agreement with previous reports of CD4 epitopes for this gp160 region in murine models (http://www.hiv.lanl.gov/content/immunology/maps/helper/gp160.html). The first targeted peptide of the C2 region (52B and 53B peptides) match previously reported CD4+ T-cell epitopes in different mouse models. However, the next C2 targeted domain aa242 to 263 (including the peptides 62/63B and 32/33BF) corresponds to a region for which at the present only human CD4+ or CD8+ T-cell epitopes have been reported (http://www.hiv.lanl.gov/content/immunology/maps).

The analysis of epitope prediction based on the probability of binding to the H-2d MHC, indicated good scores for both Class II and Class I alleles for the C2 region spanning from aa242 to 263. The higher score was obtained for the H-2Ad allele and the 62B peptide ( = 22). Interestingly, the aa changes that occurred in the corresponding BF peptide (32BF) generated a negative score association for this allele whereas the probability of association for H-2Ed allele was 16. Moreover, for Class I alleles the score values were also minor for the 32BF sequence in relation to 62B. These predictive values coincide with our experimental data, since responses found against the 32/33BF peptides were of minor magnitude, detecting a positive response in only 50% of the experiments (section 2.3 of Results).

Analysis of the peptide sequences where we detected cross-reactivity demonstrated that the aa sequence in gp160 of B or BF was identical (peptide 11) or aa changes were present at the end of the peptide (16B/13BF). On the contrary, in the situations for which a subtype specific cellular response was observed, we could verify that one aa change was enough to prevent the recognition of the peptide if it is located in a central position (peptides 62B/33BF). These results are in concordance with the properties of the TCR recognition of the peptide-MHC antigen complex. Moreover, the findings described in a recent report in which the analysis of HIV-specific CD8+ T cell responses against variant epitopes was performed [34], coincides with our results, as it was found that a single substitution in the presented epitope decreased the chance of a CTL response by 40%, and that substitutions at central positions in the peptide were particularly likely to result in abrogation of recognition. Other interesting points to highlight from this part of our analysis were the aa substitutions on the EnvBF sequence which, at least for the H-2d haplotype, have abrogated immunogenicity (Phe- Glu (FE) in envB (peptides 52B/53B) by Trp-Asp (WD) in the BF sequence); and in the case of the region in which peptides 62B and 33BF are located, the change of Arginine (R) in the EnvB sequence by Lys (K) in the EnvBF sequence notably reduced its immunogenicity. The consequences that minimal aa changes on the target peptide could have on the final T cell recognition were recently analyzed by Theodossis et.al., [35], which demonstrated that interactions between individual peptide positions impose a fine control on the conformation of pMHC-I epitopes, and that perturbation of such constraints by aa changes can lead to a previously unappreciated mechanism of viral escape.

T cell responses are regulated by different variables as available costimulation and duration of antigenic stimulation, and a clue factor is the affinity/avidity of the T cell receptor for the MHC/peptide complex. To this respect, altered peptide ligands (APL) (with substitutions in its peptide sequence) are usually recognized with a reduced affinity/avidity by the T cell receptor. In fact, it was demonstrated in a lymphocytic choriomeningitis virus (LCMV) model that cross-reactivity between APL was limited and more importantly even strongly cross-reactive cytotoxic T lymphocytes were only able to mediate moderate anti-viral protection [36]. In our study, when we analyzed whether the T cell cross-recognition has an impact on the affinity of the T cell response, we found that for the peptides analyzed (13BF and 16B) similar patterns of affinity curves were found. It must be noted that in our case, the aa substitution in the APL (the heterologous peptide) was located on the extreme of the peptide differing from the conclusion depicted for the LCMV peptide in which the substitution was on a more central position of the epitope (fourth position of a 9-mer peptide).

Quality of T-cell responses in terms of their capacity to secrete multiple cytokines is considered to have a critical role in anti-viral immunity [27]. Hence, we have evaluated if the pattern of cytokines secreted under condition of cross-recognition differs from that detected after stimulation with homologous antigen. For this, pool 1 of B peptides was employed to restimulate the splenocytes, analyzing homologous and heterologous responses in groups of animals immunized with the EnvB or EnvBF scheme respectively. No differences in the pattern of cytokines secreted between B or BF immunized animals were found. These results, along with the affinity analysis, suggest that T cell responses showing cross-recognition towards an APL containing two aa substitutions on the left extreme of the peptide do not alter appreciably these T cell properties.

When we evaluated if the combination of both single Env vaccine clades in a dual immunization scheme could induce a broad response, we found that the mixed immunization induced, in general, a wide response, covering the majority of the epitopes targeted after the individual immunizations. In other studies, where combined single-clade vaccines were applied in the Balb/c model, the authors showed that immunogenicity was limited due to multiple forms of *in vivo* immune interference [37]. In our case, we found that after the dual immunization, the response against the BF peptide located on the V3 loop was notably diminished (nearly above the limit of detection) (Fig. 7A), contrasting with the magnitude of responses detected after the single BF immunization (Fig. 4B). On the contrary, the strength of the response against the IIIB peptide (p18IIIB-I10) was maintained, as the number of SFC/10^6^ cells found in the dual group was similar to that found after the single clade B immunization (Fig. 3Bii vs Fig. 7b). This result suggested that in the presence of the two V3 peptide variants a T cell antagonistic effect was observed for the isolate IIIB over the BF variant. Similar antagonistic effects were previously reported for clade A antigen variants of Pol and Gag over the clade B antigens, also demonstrated in a Balb/c model [37]. In that study the combination the single-clade vaccines into multi-clade formulations resulted in multiple forms of *in vivo* immune interference. On the contrary, in this study with the exception of the V3 peptide, all the other peptides were targeted. Differences in the immunization schedules and combination of vectors applied may account for the discrepancies between both works, indeed other studies demonstrated that epitope immunodominance hierarchies observed upon DNA-DNA immunizations can be modified after heterologous prime-boost regimens [38]. Different studies have reported that the strategy of anatomic separation to inoculate different immunogens, can be a useful protocol to induce responses to both antigens even if one of them is immunodominant [37], [38]. Although not proven in this study, these types of protocols may be a way to circumvent the immunodominance observed for the IIIB epitope allowing the induction of a higher response against the 48BF peptide.

To extend the evaluation of the immune response induced, we also characterized cellular immune responses against the whole gp120 BAL protein (subtype B), finding certain level of cellular cross-reactivity after the BF immunization scheme. Interestingly, after the dual immunization scheme, we observed high levels of IFN-γ after stimulation of cells with gp120 BAL. On the other hand, when the antibody response (specific binding IgGs in serum) was analyzed, we found that after the BF immunization lower levels of Abs were detected against gp120 from subtype B (IIIB and BAL), compared to those in sera from mice immunized with the homologous B subtype.

The results described in this study characterized the immunogenicity of EnvBF in the Balb/c model, although cross-reactivity of murine T cells does not directly translate to humans, the underlying principles of the molecular interactions involved in triggering T cell responses are the same in both species. In fact, the Elispot assay performed with PBMCs from HIV+ persons during the primary infection stage, suggested a tendency to the recognition of Env peptides homologous to the subtype of the infecting virus.

In summary, this is the first report in which the characterization of the immunogenic and antigenic properties of Env protein from CRF12_BF in comparison with clade B is performed. In general, the majority of the cellular responses were found to be clade-specific. Interestingly, the application of a mixed immunization combining both clades (B and BF) induced a broad cellular response, in which the majority of the peptides targeted after the single clade vaccinations generated a positive response. In this group we also found significant cellular and humoral responses against the whole gp120 protein from clade B. These findings are in concordance with the theories that point toward using antigen cocktails in order to elicit increased breadth and depth of antigen-specific cellular immune responses, improving the immunologic coverage of global virus diversity. The results of this work, in conjunction with our previous published studies, suggested the convenience of the inclusion of antigens from BF variants in future vaccines for geographic regions with high prevalence of them.

## Materials and Methods

### 1. Cell lines

Stable cell lines employed in the study were: BSC-40 (epithelial cell-line derived from African green monkey kidney cells. ATCC Cat No CRL-2761); 3T3 (Balb/c embryo adherent fibroblast cells, ATCC Cat No CCL-163); HeLa (human epithelial cervix adenocarcinoma cells, ATCC Cat No CCL-2) and 293-T (epithelial cell line derived from human kidney cells, ATCC Cat No CRL-1573). Cells were maintained at 37°C in a 5% CO2 atmosphere in Dulbecco's Modified Eagle's Medium (DMEM) supplemented with 10% fetal bovine serum (FBS) (DMEM 10% FBS).

### 2. Generation of DNA and VV transfer vectors harbouring the *env*BF coding sequence

The *env* gene sequence from the HIV-1 circulating recombinant form, CRF12_BF (GenBank accession number: AF385936), was used to synthesize an *env* gene with optimized codon usage (GENEART GmbH, Regensburg, Germany). The *env*BF gene was subcloned into the VV transfer plasmid pJR101. Briefly, both the *env*-containing vector and pJR101 were digested with BamHI and NotI restriction enzymes. Then, ligation of the pJR101 backbone and the insert *env*BF was performed by using the T4 ligase (Invitrogen). In the resulting plasmid (pJR101-*env*BF), the expression of *env*BF is regulated by the VV synthetic early/late promoter (e/l) [39], being all the inserted sequences flanked by the VV hemagglutinin (HA) gene. The *env*BF insert was also subcloned into the pTARGET commercial vector (Promega) by using the same strategy in order to generate the DNA envBF expression vector. Both pJR101*env*BF and DNA*env*BF plasmids were screened for the insert and sequenced to ensure that the BF recombinant *env* sequence and restriction sites were intact. Sequencing was performed on an automatic sequencer (Applied Biosystems DNA sequencer 3100) by using the Big Dye Terminator sequencing kit (Amersham, Sweden). Nucleotide sequences were analyzed and adjusted using Sequencher 4.0.5 software (Gene Codes Co, USA).

### 3. Other DNA vectors

DNA plasmid carrying the gp120 modified for optimized codon usage (syngp120 mn V3 LAI) cloned in PCR3 (DNAenvB) as previously described [40] was a generous gift of Jürgen Hass (Munich, Germany). DNA control plasmid (DNAc) consisted of the empty pTarget plasmid. Plasmids were purified with Endo free Maxi-Prep purification kits (EndoFree Plasmid Maxi Kit, QIAgen) using pyrogen-free material and eluted in pyrogen-free TE buffer in 200 µl/column and then diluted for injection in sterile PBS.

### 4. Viruses

VV recombinants used in this study were derived from the WR strain. The VVEnvB expressing the entire *env* gene of HIV-1 strain IIIB has been previously described and employed in several studies [41], [42]. Generation of VV recombinants was performed by infection-transfection methods previously described [43] in BSC-40 cells, using the pJR*env*BF VV transfer plasmid to generate the VVEnvBF virus or the pJR empty plasmid to obtain the VVHA- control virus (VVc). For both recombinant viruses, clone purity, confirmed by PCR, was acquired after 8 passages. Amplified viral stocks were grown in BSC-40 cells and purified through 45% sucrose cushion as indicated elsewhere [44] and titration was performed in BSC-40 cells staining with crystal violet.

#### 4.1 PCR characterization of the recombinant VV*env*BF virus

Viral DNA was extracted using QIAamp DNA Mini Kit (QIAgen) from BSC-40 infected cells at a high MOI. Two types of PCR were used to verify the absence of the wtVV genome: “differential PCR” previously described [24], and the presence of the complete recombinant cassette: “integrity PCR”. In the last one integrity of the recombinant inserted gene was evidenced by the amplification of a 3 kpb, corresponding to the complete expression cassette, using two primers that target the promoter pE/L and the right extreme of the HA gene, primer pE/L: 5′ GGGTGGGTTTGGAATTA 3′ and primer HA2: 5′GATCCGCATCATCGGTGG 3′. Sequence of the syn*env*BF was corroborated by nucleotide sequencing of the DNA extracted from the 3 kpb band product obtained after the “integrity PCR”.

### 5. Western blot

Cell pellets were lysed in cold lysis buffer plus protease inhibitors Halt Protease Inhibitor Cocktail kit (PIERCE), and total protein quantities in cell lysates were determined by the Micro BCA Protein Assay Kit (PIERCE). Specified quantities of total protein from cell lysates were separated after sodium dodecyl sulfate-polyacrylamide gel electrophoresis on 10% gels, transferred to nitrocellulose membranes (Amersham), and reacted with primary rabbit anti-gp120 polyclonal, and afterwards reacted with the appropriate peroxidase-conjugated secondary antibodies. Protein expression was detected using ECL Western blotting reagents (Amersham).

### 6. Immunofluorescence

HeLa cells cultured on coverslips were infected with VVEnvBF or VV_C_ at 0.1 PFU/cell. After 18 h post-infection (p.i.) cells were washed with PBS, fixed with 4% paraformaldehyde, and permeabilized by treatment with 0.1% Triton X-100 in PBS (room temperature, 10 min). After the PBS wash, coverslips were blocked with a PBS solution containing 20% bovine serum albumin. Then, cells were incubated (1 h, at 37°C) with rabbit anti-gp120 polyclonal antibody and with mouse C3 monoclonal antibody against 14 Kda VV protein, generated in the laboratory of Dr Esteban as previously described [45]. Coverslips were washed extensively with PBS and incubated (1 h at 37°C) with secondary anti-rabbit immunoglobulin conjugated with Alexa-488 and with secondary anti-mouse immunoglobulin conjugated with Alexa-647 (Invitrogen). Antibody against the wheat germ antigen, a specific marker for Golgi structures, was included in this incubation step. After several washes with PBS, coverslips were mounted on microscope slides with Mowiol (Calbiochem). Images were obtained with a Bio-Rad Radiance 2100 confocal laser microscope.

### 7. Immunization protocols, sample collection and processing

Specific pathogen-free female (SPF) BALB/c mice (*H-2d*) six to eight weeks old were purchased from the Laboratories of the School of Veterinary Sciences, University of La Plata, Buenos Aires, and then housed in our animal facilities. All experiments were carried out in strict accordance with the recommendations in the Guide for the Care and Use of Laboratory Animals of the National Institutes of Health. The protocol was approved by the Committee of Care and Use of laboratory animals from the School of Medicine , University of Buenos Aires (Permit Number: 508/2009). Immunizations with viral vectors were given intraperitoneally (i.p.) in 200 µl of PBS, whereas DNA doses were applied in 100 µl of sterile PBS by intramuscular (i.m.) route. Doses and periods of time used in the different immunization schemes are depicted in Figures 1 and 7.

### 8. Peptides

Overlapping synthetic peptides of the EnvB consensus protein were obtained from the NIH AIDS Research and Reference Reagent Program (catalog No 9480). Lyophilized peptides were dissolved in dimethyl sulfoxide (DMSO) and stored at −20°C. Overlapping EnvBF synthetic peptides (15-mers, overlapping by 11 aa) were designed based on the sequence of the Env protein from CRF12_BF reference strain ARMA159; the same sequence employed for the construction of the DNA and VV vectors, and custom ordered from JPT Peptide Technologies (Germany). Previously characterized CD8^+^ T cell peptides: p18IIIB-I10 (RGPGRAFVTI) [46], and the VGPSNSPTF peptide for the E3L VV protein [25] were also employed. Pools of peptides covering the different constant (C) and variant (V) regions of the Env protein were formed by 7 up to 28 peptides per pool depending on the region. The epitope mapping of gp160 was carried out using the peptide-matrix based design previously described [47], in which each single peptide was present in two peptide pools. Matrix peptide pools were formed by 5 or 6 peptides at a concentration of 2 µg/ml each. Single peptides from the reactive pools were subsequently corroborated after ELISPOT assays performed with the single individual peptides at 2 µg/ml.

### 9. Murine IFN-γ ELISPOT assays

ELISPOT assays were performed using freshly isolated splenocytes as previously described [24]. Briefly, 2×10^5^ to 10^6^ cells in RPMI medium plus 10% fetal bovine serum (RPMIc) were plated in triplicate on nitrocellulose 96-well plates (MultiScreen HA plates; Millipore Corporation, Bedford) previously coated with anti-mouse IFN-γ Ab (BD™ ELISPOT Mouse IFN-γ ELISPOT Pair). Stimulus consisted of peptide pools or individual peptides. Negative controls were incubated with RPMIC plus 0.04% or 0.08% of DMSO, and cells treaated with ConA (1 µg/ml) were included as positive control. The threshold values to consider a positive response by ELISPOT was that the number of specific spots/well had to be at least 2× times the average values found in negative control wells of each group, and that after subtraction of background values, responses had to be higher than 50 SFC/million splenocytes.

Functional avidity referred to as the activation threshold in response to defined concentrations of exogenous peptide was performed following the protocols previously described [28]. Briefly, limiting peptide dilutions (from 5 to 0,0005 µM) were performed and then the peptide concentration required to induce a half-maximum IFN-γ production (number of spots) in *ex vivo* assays was determined.

### 10. Intracellular cytokine staining (ICS) of splenocytes

Splenocytes were pooled within each group, and 5×10^6^ cells were stimulated with the specified peptide pool (each peptide at a final concentration of 5 µg/ml), whole VV u.v. inactivated (at 2.5×10^7^ pfu/ml) or medium alone during 3 days. After this, cells were washed twice and the number of viable cells was calculated by trypan blue exclusion. Then, cells were dispensed in 96-well U-bottom plates (2×10^6^cells/well) with the same stimulus and the costimulatory antibody (anti-CD28 (1 ng/ml); BD Biosciences). Negative and positive controls consisted of cells stimulated with RPMIc, or PMA ionomycin (10 ng/ml phorbol myristate acetate [PMA] plus 250 ng/ml ionomycin [Sigma-Aldrich]) resepctively. After 1 h incubation at 37°C, brefeldin A (10 µg/ml) (Golgiplug, BD Biosicenes) was added and incubation was prolonged for 5 h more. Afterwards, cells were washed and stained with surface antibodies (CD3-APC, CD4-PerCP, and CD8-PerCP; BD Biosciences) for 30 min at 4°C, and then permeabilized and fixed using the Cytofix/Cytoperm kit (BDBiosciences). Living cells were identified after staining with Live/Dead APC-Cy7 (Invitrogen). After the permeabilization/fixation step, cells were stained using anti-tumor necrosis factor alpha (TNF-α) antibody labeled with PE-Cy7 (TNF-α PE-Cy7), anti-IFN-γ labeled with phycoerythrin (IFN-γ–PE) and anti-IL-2 labeled with FitC (IL-2-FitC) for 20 min at 4°C in obscurity, after two washes cells were stored at 4°C until being acquired in a BD FACSCanto flow cytometer. Data acquisition and analysis were done with the BD FACSDiva software. Instrument settings and fluorescence compensation were performed on each testing day using unstained and single-stained samples. Stimulated cells stained for surface molecules and isotype controls corresponding to intracellular markers, were included in each experiment.

### 11. T cell-specific cytokine production

Splenocytes were suspended in RPMIc and cultured in triplicate (10^6^ cells/well) into 96-well microtiter flat-bottom plates and stimulated with the indicated pool of peptides at a final concentration of 2 µg/ml each, or HIV-1_BaL_ gp120 (NIH AIDS Research and Reference Reagent Program Cat No. 4961) at 1 µg/ml. Positive controls were cells stimulated with ConA (1 µg/ml), and stimulation with medium alone or with the appropriate % of DMSO were the negative controls. After 72 h incubation at 37°C in 5% CO_2_, culture supernatants were harvested at −80°C and analyzed by ELISA for IFN-γ (BD PharMingen) following the manufacturers' instructions. The threshold values to consider a positive response was that cytokine-quantities had to be at least 2× times the average values found in control negative wells of each group.

### 12. Human PBMC samples

Peripheral blood mononuclear cells (PBMC) were isolated from whole blood of sixteen patients with primary HIV infection (time estimated from seroconversion: less than one year) and three healthy donors by Ficoll-Hypaque density gradient centrifugation (Amersham, Sweden). For viral subtyping viral RNA was extracted from plasma and used as a template to amplify the HIV-1 *pol* gene by RT-nested-PCR with posterior analysis of the amplicon nucleotide sequences [17]. Previous reports demonstrated a strong positive correlation between the viral subtype for the *pol* and *env* genes [48], [49]. The studies involving human samples were approved by the local Ethics Committee of the School of Medicine, University of Buenos Aires, and all subjects provided a written informed consent as blood sample donors.

### 13. Human IFN-γ ELISPOT assay

IFN-γ secreting cells were detected using enzyme-linked immunospot (ELISPOT) assays conducted as previously described (Turk et al., 2008, Rodríguez, et al, 2009). After the final step of the assay, plates were scanned on an ImmunoSpot reader (Cellular Technology Ltd.). Specific spots were counted using the ImmunoSpot software. Results were expressed as spot-forming cells (SFC)/10^6^ PBMC. Mean SFC background values for negative control wells were always lower than 5–10 SFC/10^6^. Thresholds for positive responses for the test wells were defined as a mean SFC greater than two times the mean SFC of the negative control wells.

### 14. Ab measurements by ELISA

The ELISA test was used to determine the presence of Abs against gp160 in serum following procedures previously described [42]. Purified gp120LAV (Protein Sciences Corp) or HIV-1_BaL_ gp120 (NIH AIDS Research and Reference Reagent Program Cat No. 4961) were employed to coat the plates 1 µg/ml. Ab detection was performed after the addition of peroxidase-conjugated goat anti-mouse IgG (Jackson ImmunoResearch Laboratories Inc), diluted 1∶5000. Reaction was developed with the peroxidase substrate TMB (Sigma) and stopped by adding 2 N H_2_SO4, absorbance was measured at 450 nm on a Multiskan Plus plate reader (Labsystems, Chicago, Ill). Samples were considered positive if optical density exceeded the mean value +3 SDs obtained for serum samples of the control group.

### 15. Bioinformatic analysis

The MHC binding affinity of peptides was predicted using web-based immunology tools: SYFPEITHI Epitope Prediction and PREDEP prediction softwares.

### 16. Data analysis

All data were expressed as the mean ± SD of triplicate determinations for each group (4 mice per group) and are representative of two to three independent experiments.

The significance of differences between the different groups of the immunized mice was determined using a two-tailed Student's *t* test assuming equal variance (GrapphPad prism4 software). A value of *P*<0.05 was considered statistically significant.

## Supporting Information

Figure S1 **Serum antibody levels against gp120B.** Serum IgG levels against recombinant gp120BAL and gp120IIIB found in the groups of mice indicated, the absorbance values shown were obtained at a serum dilution of 1/100. Results shown are representative of two independent experiments.
(TIF)

Figure S2 **Recognition of EnvBF and EnvB by HIV-infected patients.** (A) Characteristics of the HIV-1 infected patient donors of the PBMC used in the human ELISPOT assay. (B) Total anti Env T-cell responses employing EnvB and EnvBF pool peptides corresponding to the C1, C2 and V3 protein regions (using an IFN-γ ELISPOT assay as described in the Methodology). The bars show the sum of the responses against C1, C2 and V3 regions (measured as SFC/10^6^ PBMC±SD) for each patient. Background spots were subtracted. Significant differences, *p<0.05 and ** p<0.01, between BF and B responses. Cut off criteria to consider positive responses was that the number of spots in the pools stimulated wells must be >2× background values.
(PDF)
