# Supplementary figures and images for: T-Cell Immune Responses Against Env from CRF12_BF and Subtype B HIV-1 Show High Clade-Specificity that Can Be Overridden by Multiclade Immunizations
Source: PLoS One. 2011 Feb 18;6(2):e17185. doi: 10.1371/journal.pone.0017185 (PMC3041790; doi:10.1371/journal.pone.0017185)

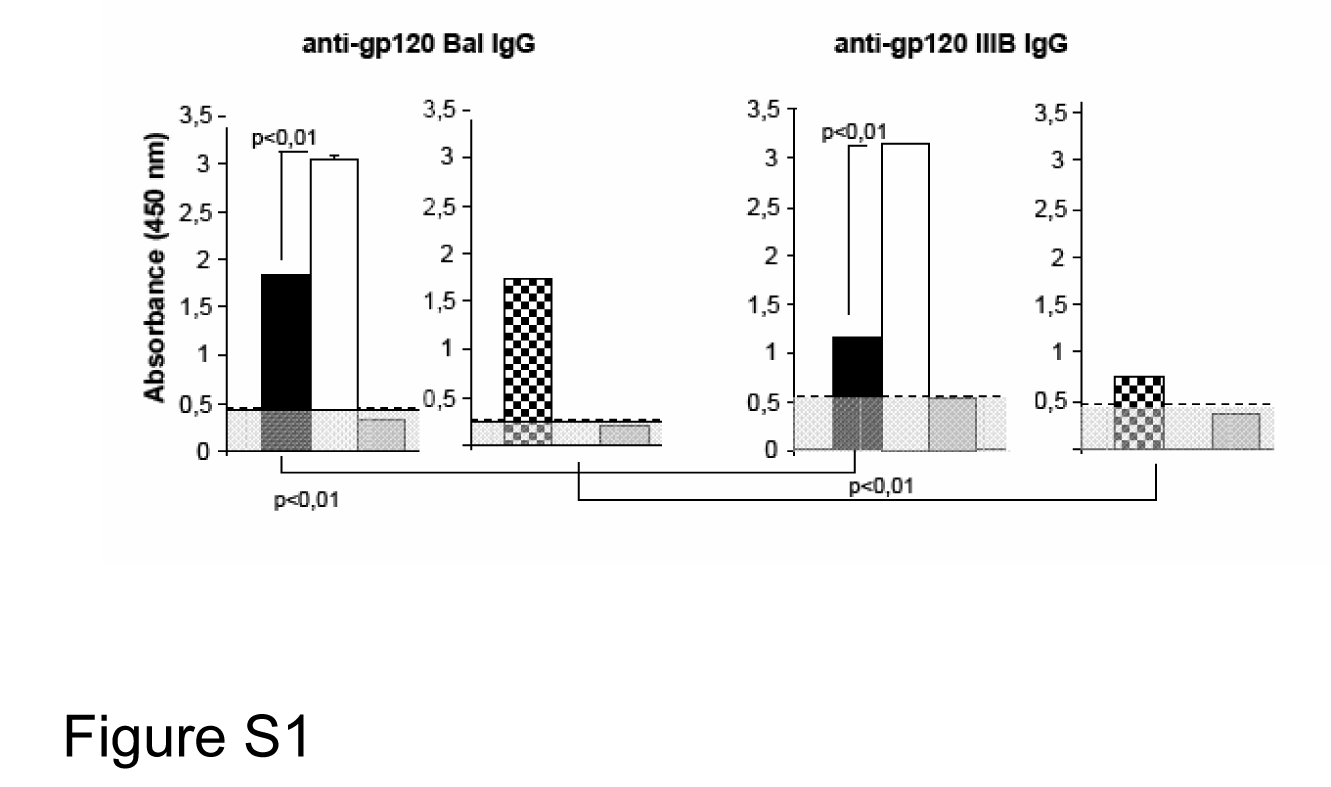

Supplement: Figure S1 — Serum antibody levels against gp120B. Serum IgG levels against recombinant gp120BAL and gp120IIIB found in the groups of mice indicated, the absorbance values shown were obtained at a serum dilution of 1/100. Results shown are representative of two independent experiments. (TIF) [file pone.0017185.s001.tif]
