# Supplementary material for: T-Cell Immune Responses Against Env from CRF12_BF and Subtype B HIV-1 Show High Clade-Specificity that Can Be Overridden by Multiclade Immunizations
Source: PLoS One. 2011 Feb 18;6(2):e17185. doi: 10.1371/journal.pone.0017185 (PMC3041790; doi:10.1371/journal.pone.0017185)

A

Characteristics of the human blood donors

Donor N°	Gender	Viral Load (VL)[a] log10	CD4+ (cells/µl)[b]	CD8+ (cells/µl)[b]	Viral Subtype	Anti-Env Response
**391**	**M**	**>5.7**	**140**	**431**	**B**	**Positive**
078	M	5.233	381	437	B	Negative
471	F	>5.7	379	1484	B	Negative
**834**	**M**	**5.124**	**662**	**1123**	**B**	**Positive**
718	F	2.88	983	874	B	Negative
**126**	**M**	**4.28**	**653**	**864**	**B**	**Positive**
814	M	4.669	415	1184	B	Negative
488	M	5.69	536	1315	BF	Negative
134	F	>5.7	352	787	BF	Negative
**732**	**M**	**3.44**	**403**	**706**	**BF**	**Positive**
**183**	**M**	**ND**	**1022**	**1598**	**BF**	**Positive**
**690**	**F**	**4.898**	**472**	**617**	**BF**	**Positive**
946	M	2.87	619	871	BF	Negative
069	F	3.473	1000	1085	BF	Negative
428	M	4.7	895	1667	BF	Negative
757	M	4.94	362	576	BF	Negative
001	F	-	-	-	NI	Negative
002	F	-	-	-	NI	Negative
003	M	-	-	-	NI	Negative

[a] Viral load (VL) calculated by the Versant HIV-1 RNA 3.0 assay (Bayer AG). The detection threshold was 50 RNA copies/ml (1.7 log10).
[b] Flow cytometry double platform (Epics XL; Coulter).
ND not-determined.
NI non-infected.

B

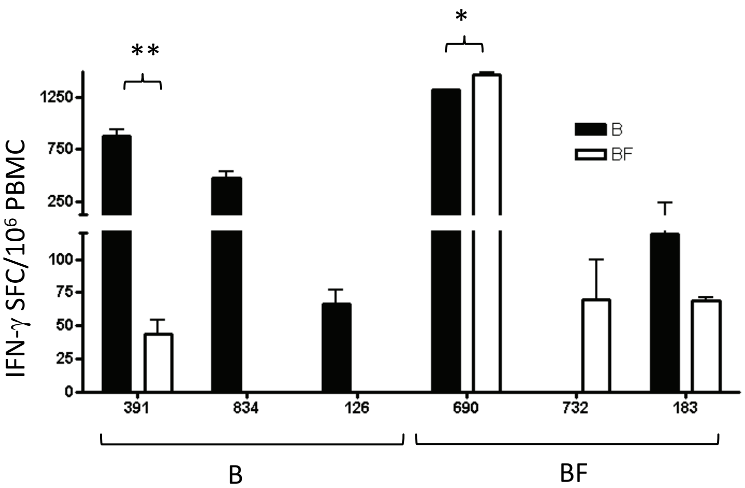

Supplement: Figure S2 — Recognition of EnvBF and EnvB by HIV-infected patients. (A) Characteristics of the HIV-1 infected patient donors of the PBMC used in the human ELISPOT assay. (B) Total anti Env T-cell responses employing EnvB and EnvBF pool peptides corresponding to the C1, C2 and V3 protein regions (using an IFN-γ ELISPOT assay as described in the Methodology). The bars show the sum of the responses against C1, C2 and V3 regions (measured as SFC/106 PBMC±SD) for each patient. Background spots were subtracted. Significant differences, *p<0.05 and ** p<0.01, between BF and B responses. Cut off criteria to consider positive responses was that the number of spots in the pools stimulated wells must be >2× background values. (PDF) [file pone.0017185.s002.pdf]
